# Intra-abdominal pressure measurement using the FoleyManometer does not increase the risk for urinary tract infection in critically ill patients

**DOI:** 10.1186/2110-5820-2-S1-S10

**Published:** 2012-07-05

**Authors:** Nele Desie, Alexandra Willems, Inneke De laet, Hilde Dits, Niels Van Regenmortel, Karen Schoonheydt, Martine Van De Vyvere, Manu LNG Malbrain

**Affiliations:** 1 Department of Intensive Care, Ziekenhuis Netwerk Antwerpen (ZNA) Stuivenberg, Lange Beeldekensstraat 267, B-2060 Antwerpen, Belgium; 2 Department of Microbiology, Ziekenhuis Netwerk Antwerpen (ZNA) Stuivenberg, Antwerpen, Belgium

**Keywords:** intra-abdominal pressure, abdominal compartment syndrome, abdominal hypertension, FoleyManometerLV, intensive care, intravesical pressure, intrabladder pressure, urinary tract infection

## Abstract

**Objective:**

The aim of this study was to determine whether intra-abdominal pressure (IAP) monitoring using the FoleyManometer (Holtech Medical, Charlottenlund, Denmark) increases the risk of urinary tract infection (UTI).

**Design:**

A retrospective database review was conducted.

**Setting:**

The study was conducted in the 12-bed medical intensive care unit of ZNA Stuivenberg Hospital (Antwerp, Belgium), a tertiary hospital.

**Patients:**

There were 5,890 patients admitted to the medical intensive care unit of which 1,097 patients underwent intrabladder pressure (IBP) monitoring as estimate for IAP.

**Interventions:**

Crude and adjusted UTI rates were compared among patients undergoing IAP measurements with three different intrabladder methods: a modified homemade technique, a FoleyManometer with 35 ml reservoir, and a FoleyManometer low volume (FoleyManometerLV) with less than 10 ml priming volume.

**Measurements and results:**

Four consecutive time periods of 24 months were defined and compared with regard to IAP measurement: period 1 (2000-2001), during which IAP monitoring was not used routinely (which serves as a control group), was compared with period 2 (2002-2003), using a modified homemade technique; period 3 (2004-2005), introducing the FoleyManometer; and finally period 4 (2006-2007), in which the FoleyManometerLV was introduced. The incidence of IBP measurements increased from 1.4% in period 1 to 45.4% in period 4 (*p *< 0.001). At the same time, the Simplified Acute Physiology Score (version 2) (SAPS-II) increased significantly from 24.4 ± 21.5 to 34.9 ± 18.7 (*p *< 0.001) together with the percentage of ventilated patients from 18.6% to 40.7% (*p *< 0.001). In total, 1,097 patients had IAP measurements via the bladder. The UTI rates were adjusted for disease severity by multiplying each crude rate with the ratio of control versus study patient SAPS-II probability of mortality. Crude and adjusted UTI rates per 1,000 catheter days (CD) were on average 16.1 and 12.8/1,000 CD, respectively, and were not significantly different between the four time periods.

**Conclusions:**

Intrabladder pressure monitoring as estimate for IAP either via a closed transducer technique or the closed FoleyManometer technique seems safe and does not alter the risk of UTI in critically ill patients.

## Introduction

Intra-abdominal hypertension (IAH) and abdominal compartment syndrome (ACS) have been shown to contribute to organ dysfunction and mortality in critically ill patients. Diagnosis relies on intra-abdominal pressure (IAP) measurement, as clinical estimation and abdominal perimeter are poorly correlated with the actual IAP [1-5].

Different techniques have been developed, either measuring IAP directly or indirectly (via stomach, bladder, rectum, or inferior vena cava) [2,6-8]. Intrabladder pressure (IBP) monitoring is considered the method of choice for indirect IAP measurement due to its accuracy and relative ease [9-11]. IBP is measured through the patient's indwelling urinary Foley catheter, utilizing the bladder wall as a passive transducing membrane.

Although the benefits of IBP monitoring in the diagnosis, prevention, and management of IAH/ACS have been demonstrated, some clinicians remain reluctant to institute this monitoring technique out of concern for increasing the patient's risk of device-related nosocomial urinary tract infection (UTI) [12]. There are only few scientific data to support or refute this theory. Cheatham et al. published a retrospective study about the risk of UTI in relation to IBP monitoring using a closed transducer technique, and he concluded that IBP monitoring did not increase the risk of UTI in 122 patients [12]. Ejike et al. found similar results in a prospective observational study in critically ill children [13]. On the contrary, Duane et al. demonstrated a greater risk of UTI with bladder pressure measurements, this time using an open technique [14]. The FoleyManometer (Holtech Medical, Charlottenlund, Denmark, http://www.holtech-medical.com) is a relatively new device for IBP measurement using the height of the urine column as IAP estimate, with the advantage that it does not require a pressure transducer and can be used outside the ICU [15]. Because the device uses the patient's own freshly produced urine to measure IAP and not saline like the other techniques do, the risk of device-related UTI could either be higher (by the fact that urine is re-introduced) or lower (since freshly produced urine is by definition sterile, the FoleyManometer is a closed system and less manipulations are needed). To our knowledge, there are no published data so far on the incidence of UTI using the FoleyManometer. The results of this study were presented as an oral presentation at the 4th World Congress for the Abdominal Compartment Syndrome held in Dublin, Ireland (http://www.wcacs.org, June 2009) [16].

## Materials and methods

### Patients

This is a retrospective cohort study conducted on the electronic patient files of all 5,890 patients admitted to the 12-bed medical ICU of a tertiary hospital (Ziekenhuis Netwerk Antwerpen, ZNA Stuivenberg Hospital, Antwerp, Belgium) during an 8-year period stretching from January 2000 to December 2007. Using the electronic ICU patient database (developed with FileMaker Pro 7.0 software, FileMaker, Inc., Santa Clara, CA, USA), patient demographics, number of mechanical ventilation days, urinary catheter days (CD), number of IBP measurements, and patient outcome were collected. Severity of illness was evaluated using the Simplified Acute Physiology Score (version 2) (SAPS-II). Patient data were accessed via the database program and exported to an Excel worksheet (Microsoft, Redmond, WA, USA). All patients were cultured according to the standing ICU protocol. The study was approved by the local institutional review board without need for informed consent due to the retrospective nature of the analysis.

### Intrabladder pressure measurements

**Figure 1:**
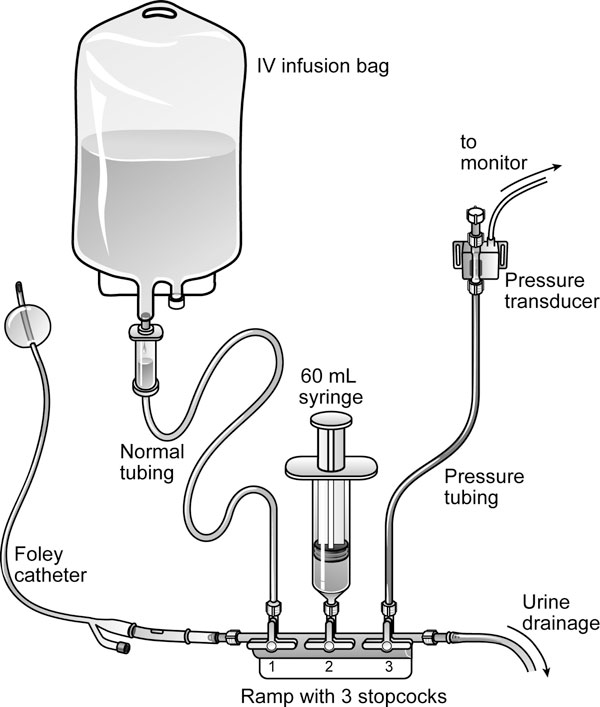
**Modified method for intrabladder pressure monitoring as described by Malbrain (adapted from **[3]**with permission)**. Setup: - Using sterile scissors, the drainage tubing is cut 40 cm after the culture aspiration port after desinfection. - A ramp with three stopcocks is connected to a conical connection piece at each side with a male/male adaptor and inserted. - A standard intravenous (IV) infusion set is connected to a bag of 500 ml of saline and attached to the first stopcock. - A 60-ml syringe is connected to the second stopcock, and the third stopcock is connected to a pressure transducer via rigid pressure tubing. - The system is flushed with normal saline. Method of measurement: - Patient in supine position. - Zero pressure module at the midaxillary line at the level of the iliac crest (mark for future reference) by turning the proximal stopcock onto the air and the transducer. - At rest, the three stopcocks are turned 'off' to the IV bag, the syringe, and transducer giving an open way for urine to flow into the urometer - To measure IBP, the urinary drainage tubing is clamped distal to the ramp, and the third stopcock is turned 'on' to the transducer and the patient - The first stopcock is turned 'off' to the patient and 'on' to the IV infusion bag; the second stopcock is turned 'on' to the IV bag and the 60-ml syringe. - Aspirate 20-25 ml of saline from the IV bag into the syringe. - The first stopcock is turned 'on' to the patient, and the 20-25 ml of normal saline is instilled in the bladder - The first and second stopcocks are then turned 'on' to the patient and thus turned 'off' to IV tubing and the syringe. - The third stopcock already being turned 'on' to the transducer and patient allows then immediate IBP reading on the monitor.

**Figure 2:**
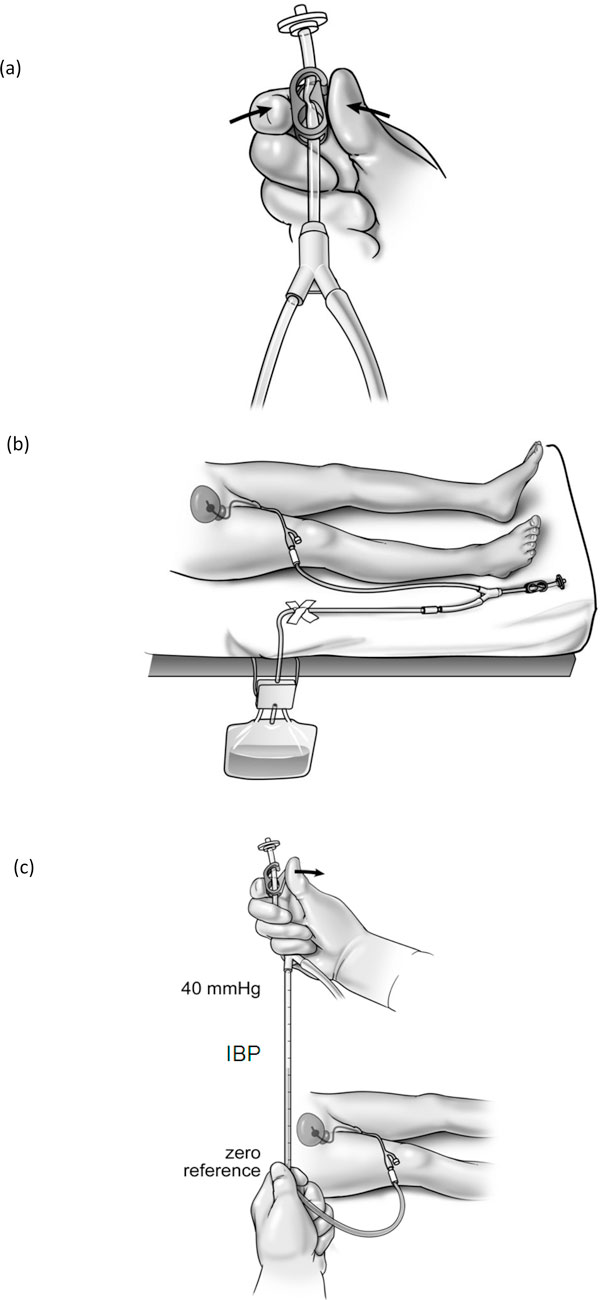
**Intrabladder pressure monitoring with the FoleyManometerLV**. This technique that uses the patient's own urine as pressure transmitting medium is a simple, reliable, and cost-effective clinical tool. Based on a modified version of the IAP monitoring technique described by Malbrain [3], the disposable FoleyManometer provides a closed sterile circuit which connects between the patient's Foley catheter and the urine collection device. Each IAP determination takes about 10 s, and no subsequent correction of urine output is required. The technique uses a low bladder infusion volume, has a needle-free sampling port, and can measure IAP in a range from 0 to 40 mmHg. **A **Initial setup: - Open the FoleyManometer LV pouch and close the tube clamp. - Place the urine collection device under the patient's bladder and tape the drainage tube to the bed sheet. - Insert the FoleyManometer between catheter and drainage device. - Prime the FoleyManometer with 20 ml of sterile saline through its needle-free injection/sampling port. - Prime only once at initial setup **B **Urine drainage: - Let the urine drain in between IBP measurements. - Urine sampling from the needle-free port is facilitated by temporarily opening the red clamp. Remember to close clamp afterward. - Avoid a U-bend of the large urimeter drainage tube (which will impede urine drainage). - Replace the FoleyManometer whenever the Foley catheter or the urine collection device is replaced, or at least every 7 days. **C **Intravesical pressure monitoring: - Place the '0-mmHg' mark of the manometer tube at the midaxillary line at the level of the iliac crest (mark for future reference) and elevate the filter vertically above the patient. - Open the bio-filter clamp and read IBP (end-expiration value) when the meniscus has stabilized after about 10 s. - Close clamp after IBP measurement and place the FoleyManometer in its drainage position.

According to the number of patients with IAP monitoring and the used IAP measurement technique, the 8-year period could be divided into four distinct time periods. Initially, only some patients showing one of the symptoms of elevated IAP (e.g., abdominal distension, hypercapnia, hypoxemia refractory to increasing inspired oxygen fractions, and positive end-expiratory pressure,...) or patients in which the attending physician had a suspicion of elevated IAP without any other symptoms underwent IBP monitoring (period 1, 2000-2001). This period in which IAP was measured in 28 out of 2,046 patients served as the control group. Later, in period 2 (2002-2003), IBP monitoring was done more systematically resulting in the measurement of IAP in all mechanically ventilated patients from the beginning of 2004. The IBP was then measured every 8 h. In period 2 (as well as the few patients in period 1), a modified Cheatham technique (with a closed stopcock system and instillation of 50 ml sterile saline in the bladder) was used to measure IAP (Figure 1). This technique has been described into detail previously [3]. In period 3 (2004-2005), the FoleyManometer with a 35-ml reservoir (Holtech Medical, Charlottenlund, Denmark, http://www.holtech-medical.com) was introduced as the standard technique, and finally, in period 4 (2006-2007), the newer version FoleyManometer Low Volume (FoleyManometerLV, Holtech Medical, Charlottenlund, Denmark, http://www.holtech-medical.com), with less than 10-ml infusion volume, was used in all patients (Figure 2).

The standard Foley catheter used was the 14-Fr Bard Biocath (CR Bard, Medical Division, Covington, GA, USA). Urine drainage tubing, collection bags, and FoleyManometers were replaced every 7 days.

### Definitions

Urine analysis and urine cultures (UC) were obtained two times a week (standard on Monday and Thursday) from mechanically ventilated patients and additionally in any patient that developed a fever of > 38.5°C or macroscopically grossly purulent urine. As previously described by Cheatham, UTI was defined by microbiological culture documentation of more than 100,000 colony-forming units per high power field of either a specific bacterium or fungus together with significant pyuria defined as either more than five leucocytes per high power field or more than 25 leucocytes per microliter. Infection rates were calculated using the Centers for Disease Control National Nocosomial Infections Surveillance System definitions and criteria [17]. Each 24-h period that a patient had a urinary catheter in place (or > 12 h portion thereof) was considered to be one 'catheter day'. Since virtually all patients had a Foley in place during their ICU stay, the number of CD was considered equal to the number of ICU days. Data were collected on the total number of UC taken as well as the number of UC per patient, since one can assume that the more UC are taken, the more likely the chance that a positive UC will be identified. The crude UTI risk was defined as the chance of identifying a positive UC, or thus the number of UTI divided by the total number of patients. The adjusted UTI risk was defined as the number of UTI divided by the number of UC taken. Infection rates are reported as the number of infections per 1,000 CD. For statistical analysis, period 1 was used as a control group (since the incidence of IAP measurement was only 1.4% and the other periods were compared to each other and the control group). The crude UTI rate was defined as the number of UTI/period/1,000 CD, and this was analyzed in relation to the 1,097 patients that underwent IAP measurements. The UTI rates were then adjusted for disease severity, and the adjusted UTI rate was defined as the crude UTI rate multiplied by the ratio of control (group 1) versus study (groups 2-4) patients SAPS-II probability of mortality and compared using the *z*-approximation for independent proportions.

### Statistical analysis

Descriptive statistics are presented as mean ± standard deviation. Dichotomous variables were compared using the chi-squared test, while continuous variables were compared using Student's *t *test. Statistical significance was defined at two-tailed *p *value levels of 0.05.

## Results

Between January 2000 and December 2007, 5,890 patients were admitted to the medical intensive care unit; in 1,097 (18.6%) of these patients, IBP was monitored as estimate for IAP. Data regarding patient demographics, severity of illness, and outcome during the four periods are presented in Table 1. The number of patients admitted decreased significantly over the four periods while mean ICU length of stay increased from 4.1 to 7.1 days. Severity of illness, number of mechanical ventilation days, and mortality significantly increased over the four periods (*p *< 0.001).

**Table 1:** Demographic data

	Period 1	Period 2	Period 3	Period 4	Total	*p *value (period 1 vs 4)
			
	Control group	Modified Cheatham technique	FoleyManometer	FoleyManometerLV		
			
	2000-2001	2002-2003	2004-2005	2006-2007		
Patients (*n*)	2,046	1,480	1,261	1,103	5,890	-
Age (years)	66 ± 17.5	65 ± 16.2	65 ± 16.3	63 ± 18.7	65 ± 17.1	NS
SAPS-II	24.4 ± 21.5	30.1 ± 20.4	32.4 ± 18.7	34.9 ± 18.7	29.8 ± 20.4	< 0.001
ICU days	8,045	7,265	7,584	7,161	30,055	-
MV days	2,988	3,343	3,192	3,785	13,308	-
%MV (days)	37.1%	46%	42.1%	52.9%	44.32%	< 0.001
IAP patients	28 (1.4%)	146 (9.9%)	422 (33.5%)	501 (45.4%)	1,097 (18.6%)	< 0.001
MV patients	381 (18.6%)	404 (27.3%)	418 (33.1%)	449 (40.7%)	1,652 (28.1%)	< 0.001
ICU stay (days)	4.1 ± 7.3	4.3 ± 7.6	5.1 ± 8.7	7.1 ± 16.6	5.3 ± 11	< 0.001
Predicted mortality	16.5% ± 23.8	20.5% ± 25	21.2% ± 25.6	24.3% ± 26.3	20.1% ± 25.2	< 0.001
ICU mortality	11%	12.7%	15.2%	17.4%	13.5%	< 0.001

Severity of illness and outcome of critically ill patients during the four time periods. IAP, intra-abdominal pressure; ICU, intensive care unit; MV mechanical ventilation; SAPS, Simplified Acute Physiology Score.

Data regarding the crude and adjusted rate of UTI in the different periods are shown in Table 2. The total number of UC taken and the number of cultures per patient increased from 915 to 1,896 and from 0.5 to 1.7, respectively. The number of positive UC as a ratio to the number of UC taken decreased in period 4 as compared to the control group from 12.5% to 9% (*p *= 0.007).

**Table 2:** Risk and rate of urinary tract infections during the four study periods

	Period 1	Period 2	Period 3	Period 4	Total
		
	Control group	Modified Cheatham technique	FoleyManometer	FoleyManometerLV	
		
	2000-2001	2002-2003	2004-2005	2006-2007	
UC	915	1,062	1,568	1,896	5,441
UC/patient	0.4	0.7	1.2	1.7	0.9
POS UC	114	119	204	171	608
POS UC/UC sample, %	12.5	11.2	13	9*	11.2
UTI	102	106	133	140	481
UTI risk (CR), %	5.0	7.2	10.5	12.7°	8.2
UTI risk (ADJ), %	11.1	10.0	8.5	7.4	8.8
UTI/1,000 CD (CR)	12.7	14.6	17.5^§^	19.6^#^	16.1
UTI/1,000 CD (ADJ)	12.7	11.7	13.6	13.3	12.8

ADJ, adjusted; CD, catheter days; CR, crude rate; POS, positive (bacteriuria); UC, urine culture; UTI, urinary tract infection = positive UC with more than five leucocytes per high power field or more than 25 leucocytes per microliter; UTI risk (CR), the number of UTI divided by the total number of patients; UTI risk (ADJ), the number of UTI divided by the number of UC taken; UTI/1,000 CD (CR), the number of UTI/period/1,000 CD; UTI/1,000 CD (ADJ), the crude UTI rate multiplied by the ratio of control (group 1) versus study (groups 2-4) patients SAPS-II probability of mortality. *p value 0.007, 0.06, and < 0.001 when period 4 was compared to period 1 (control), period 2 and period 3, respectively, regarding number of positive UC with regard to total number of UC; °*p *value 0.039 and 0.035 when period 4 was compared to period 1 and 2, respectively; ^#^*p *value < 0.001 and 0.011 when period 4 was compared to period 1 and 2, respectively; ^§^*p *value 0.007 when period 3 was compared to period 1.

Overall, the probability to identify a UTI per patient studied (or thus the crude UTI risk) was on average 8.2% but increased significantly in periods 3 and 4 (10.5% and 12.7%, respectively), when compared to the 5% in the control group (*p *= 0.039 and *p *= 0.035, respectively). The adjusted UTI risk, also taking into account the number of UC taken, however decreased in period 4 (7.4%) compared to the other periods and the control group (11.1%), resulting in non-significant differences between the four time periods.

The crude UTI rate per 1,000 CD was on average 16.1 but was significantly higher in periods 3 (17.5) and 4 (19.6) when compared to the control (12.7) group (*p *= 0.007 and < 0.001, respectively). However, the severity adjusted UTI rate per 1,000 CD was on average 12.8 and was not significantly different between the four time periods.

**Figure 3:**
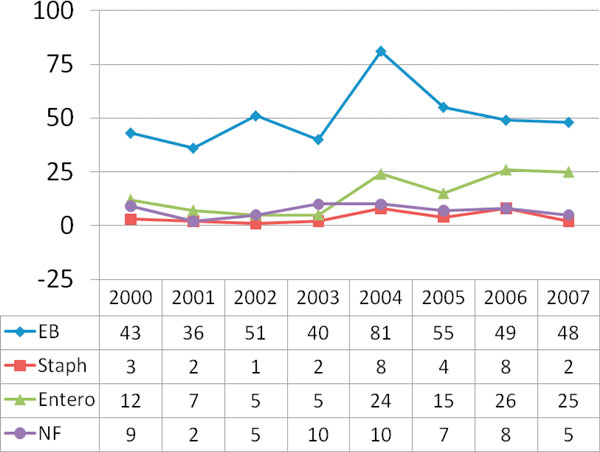
**Evolution of pathogens identified in urine cultures during the different time periods**. EB, enterobacteriaceae; NF, non-fermenters (e.g., pseudomonas); Staph, staphylococci; Entero, enterococci.

The pathogens identified in the 608 positive UC were predominantly enterobacteriaceae, enterococci, non-fermenters, and, rarely, staphylococci, as shown in Figure 3. In 2004, a local epidemic with enterobacteriaceae was noted not only in urine cultures but also in endobronchial/tracheal aspirates and blood cultures.

## Discussion

IAP has gained interest in a wide variety of patient populations since IAH and ACS have been recognized as a major cause of potentially life-threatening end-organ dysfunction. As IAP increases, the physiology of multiple organ systems is affected leading to inadequate organ perfusion and tissue oxygenation, multiple organ failure, and death. IBP monitoring has been recommended by the World Society on Abdominal Compartment Syndrome http://www.wsacs.org as the method of choice for indirect measurement of IAP due to its accuracy and relative ease of measurement [10,11].

The original method for IBP monitoring was described by Kron and co-workers and required the patient's urinary catheter to be disconnected [18]. This leads to justified fears for device-related UTI. The modified technique proposed by Cheatham still used sterile saline instillation into the bladder but maintained the patient's urinary catheter as a closed system, which put to rest some of the concerns relating to UTI [19]. The FoleyManometer is also a closed sterile system but uses freshly produced urine as IAP transmitting medium and measures the height of the fluid/urine column in an especially designed drainage tube with a 35-ml reservoir. After several reports stating that lower instillation volumes can and should be used for IBP measurements in order to avoid overestimation of IAP, a new FoleyManometerLV was designed with less than 10-ml infusion volume [11,20,21]. The re-instillation of the patient's own urine which may have been in the drainage tube for some time raised even more concerns about the risk for UTI than the previous IBP measurement techniques. Our study is, to our knowledge, the first to examine the risk for UTI using urine as a transmitting medium for IBP measurement.

Before this publication, Cheatham et al. published already a study on UTI risk in relation to IBP monitoring using other devices [12]. They found that IBP monitoring using a closed transducer technique with sterile saline instillations is safe and does not increase the risk of UTI. Ejike et al. found similar results in a prospective observational study in critically ill children, also using a closed technique with sterile saline installations [13]. On the other hand, Duane et al. demonstrated a greater risk of UTI with bladder pressure measurements using an open technique in which the Foley catheter was compromised through insertion of an 18-gauge needle and disconnection of the Foley catheter to allow instillation of 50 ml of saline into the bladder [14]. We confirmed that the UTI rate, when adjusted for disease severity, remained unchanged with or without IBP monitoring using different devices, all being closed systems. There was a trend toward higher adjusted UTI rates using the FoleyManometer with larger instillation volumes that was used during period 3, but this was not statistically significant when compared to the other groups. With the newer FoleyManometerLV, the trend toward higher adjusted UTI rates disappeared, and thus, this is a safe technique.

The UTI rates per 1,000 CD reported in this study are higher than those reported by Cheatham who found 10.4 crude and 7.9 adjusted UTI/1,000 CD in the patient population that underwent IAP monitoring versus 6.5 in the control group. We observed 12.7 in our control group, and this could be related to the fact that CD in our study coincided with ICU stay, so it could be possible that some patients kept their Foley catheters after ICU discharge leading to actual longer CD (that were not taken into account) and thus overestimation of UTI risk. In a large study on 2,644 ICU patients, van der Kooi et al. found a UTI incidence of 8% (versus 8.8% in our study) and 9 UTI/1,000 CD (versus 16.1 crude and 12.8 adjusted in our study) [22]. In another study on 337 adult ICU patients, the crude risk for UTI was 14/1,000 CD, which is close to what we observed [23].

One could state that the more UC taken, the greater the chance of identifying a positive UC. But although the total number of UC taken and thus the number of UC per patient increased during our observation, there was no increase in the UTI rates during period 4.

The strong points of our study, although still retrospective and purely observational in nature, are the presence of a historic control group, allowing comparison of subsequent groups versus a 'baseline' UTI rate, and, more importantly, the standardized approach to both IAP monitoring and urine sampling. IAP monitoring is used in all mechanically ventilated patients in our ICU, and cultures are taken at least twice a week or more frequently if clinically indicated.

There are also some important and obvious limitations to our study. First of all, there is the retrospective design leading to missing data on antibiotic use and other infectious foci, among other problems. Secondly, our patient population evolved significantly over the 8-year time period. Disease severity (SAPS-II score), ICU length of stay, number of ventilation days, and ICU mortality increased significantly over the four time periods. To correct for this evolution, crude UTI rates were adjusted for disease severity, but naturally, this does not correct for all possible sources of bias in the study. Third, potential etiological factors for the development of IBP monitoring-associated UTI were not considered. The duration of urinary catheterization could be different in the four groups, although this was determined by the standard ICU procedures as stated in the methods (urine drainage tubing, collection bags, and FoleyManometers were replaced every 7 days). Fourth, due to the epidemiologic and observational nature of the study looking at global incidences, we could not look for individual factors predictive for UTI or outcome by multiple logistic regression analysis.

## Conclusion

In summary, IBP monitoring with a closed transducer technique or with the FoleyManometerLV, as estimate for IAP, does not have an influence on the risk of UTI in critically ill patients.

## Abbreviations

ACS: abdominal compartment syndrome; CD: catheter days; FoleyManometerLV: FoleyManometer low volume; IAH: intra-abdominal hypertension; IAP: intra-abdominal pressure; IBP: intrabladder pressure; ICU: intensive care unit; IV: intravenous; MV: mechanical ventilation; SAPS: Simplified Acute Physiology Score; UC: urine culture; UTI: urinary tract infection.

## Competing interests

MM is founding president and treasurer of the World Society on Abdominal Compartment Syndrome and a member of the medical advisory board of Holtech Medical (Charlottenlund, Denmark), an IAP monitoring company. The other authors declare that they have no competing interests.

## Authors' contributions

ND, AW, IDL, NVR, KS, HD, and MM planned the study and were responsible for the design, coordination, and drafting the manuscript. MV participated in the study design and collected all microbiology data. ND, AW, and MM performed the statistical analysis and helped to draft the manuscript. All authors read and approved the final manuscript.
